# Distribution of flagella secreted protein and integral membrane protein among *Campylobacter jejuni *isolated from Thailand

**DOI:** 10.1186/1757-4749-3-11

**Published:** 2011-07-12

**Authors:** Piyarat Pootong, Oralak Serichantalergs, Ladaporn Bodhidatta, Frédéric Poly, Patricia Guerry, Carl J Mason

**Affiliations:** 1 Enteric Diseases Department, Armed Forces Research Institute of Medical Sciences, Bangkok, 10400, Thailand; 2 Enteric Diseases Department, Naval Medical Research Center, Silver Spring, MD 20910, USA

## Abstract

**Background:**

*Campylobacter jejuni*, a gram-negative bacterium, is a frequent cause of gastrointestinal food-borne illness in humans throughout the world. There are several reports that the virulence of *C. jejuni *might be modulated by non-flagellar proteins that are secreted through the filament. Recently, FspA (Flagella secreted proteins) have been described. Two alleles of *fspA *(*fspA1 *and *fspA2*) based on sequence analysis were previously reported and only the *fspA2 *allele was found in Thai isolates. The aim of this study is to analyze the deduced amino acid sequences *fspA *and the adjacent putative integral membrane protein from 103 Thai *C. jejuni *isolates.

**Results:**

A total of 103 representative *C. jejuni *isolates were amplified by PCR for the *fspA *gene and the adjacent integral membrane protein gene. Two PCR product sizes were amplified using the same primers, an approximately 1600-bp PCR product from 19 strains that contained *fspA *and integral membrane protein genes and an approximately 800-bp PCR product from 84 strains that contained only the *fspA *gene. DNA sequencing was performed on the amplified products. The deduced amino acid sequences of both genes were analyzed separately using CLC Free Workbench 4 software. The analysis revealed three groups of FspA. Only FspA group 1 sequences (19/103) (corresponding to *fspA1*) consisting of 5 subgroups were associated with the adjacent gene encoding the integral membrane protein. FspA group 2 was the largest group (67/103) consisting of 9 subgroups. FspA group 2p (17/103) consisting of 7 subgroups was found to contain stop codons at a position before the terminal 142 position.

**Conclusions:**

This study reveals greater heterogeneity of FspA (group 1, 2 and 2p) among Thai *C. jejuni *isolates than previously reported. Furthermore, the subgroups of FspA groups 1 were associated with groups of integral membrane protein. The significance of these different FspA variants to virulence requires further study.

## Background

*Campylobacter jejuni *is a major cause of gastroenteritis worldwide especially in children, travelers, military personnel deployed to developing countries. Although these pathogens are generally considered invasive, the level of invasion of intestinal epithelial cells *in vitro *varies among strains [1]. Despite the high incidence of human disease and multiple genome sequences [2-5], understanding about the pathogenesis of diarrheal disease at the molecular level is limited. Genomic studies have indicated that *C. jejuni *strains lack specialized type III secretion systems that are essential to virulence of many other enteric pathogens [6,7]. There are several reports that flagella can function to secrete non-flagellar proteins that might modulate virulence.

The Cia (*Campylobacter *invasion antigen) proteins [8-10] and FlaC (Flagellin C protein) [11] are non-flagellar proteins that are secreted through the filament. Recently, a third secreted protein, FspA (Flagella secreted protein) has been described [12] and additional candidates have also been reported recently [13]. Similar to FlaC, FspA is a small acidic protein is secreted into the supernatant of broth-grown bacteria. Two alleles of *fspA *(*fspA1 *and *fspA2*) based on sequence analysis were found in previous reports that examined strains from diverse geographical areas. The entirety of the Thai isolates tested in this previous study contained *fspA2 *alleles. Recombinant proteins encoded by an *fspA1 *allele from strain 81-176 and by an *fspA2 *allele from strain CG8486, a clinical isolate from Thailand [5] were studied previously. These studies revealed that recombinant FspA2 bound to intestinal epithelial cells *in vitro *and also induced apoptosis, whereas recombinant FspA1 did not [12]. Thus, only one form of the FspA protein appeared toxic to eukaryotic cells, but the mechanism and significance to virulence remains to be determined. Moreover, the study of immunogenicity and protective efficacy of FspA1 and FspA2 were compared in a mouse intranasal model. Immunization with FspA1 resulted in higher protection against homologous challenge with 81-176, which expresses the homologous FspA1, and CG8486, which expresses FspA2; immune protection with FspA2 was less robust against both strains [14]. Genomes presenting *fspA2 *allele (encoded from Cj0859c gene in reference strain NCTC11168) have been reported to systematically lack the adjacent gene Cj0860, a putative integral membrane protein. In contrast, the *fspA1 *allele was adjacent to the integral membrane protein gene [12]. However, no functional relation has been reported between these two proteins. Additionally, another report [15] showed FspA variants and MLST associations among human, poultry and bovine *Campylobacter jejuni *strains.

A significant high isolation rate of *C. jejuni *in Thailand and potential role of the FspA protein as one of vaccine candidate lead us to further characterize the deduced amino acid sequences *fspA *and the adjacent putative integral membrane protein from additional Thai *C. jejuni *isolates.

## Results and Discussion

The 103 *C. jejuni *isolates representing clusters from a previous PFGE (Pulsed Field Gel Electrophoresis) cluster analysis of *C. jejuni *isolates from the diarrhea studies on adults in Thailand were selected for this study [16]. We investigated the variation of FspA and integral membrane protein by DNA sequencing of PCR products and analyzed their deduced amino acid sequences.

### PCR and sequencing

Cj0859c and Cj0860, if present, were amplified from DNA samples of the strains. Approximate 1600-bp PCR products containing both genes were obtained from 19 strains and approximate 800-bp PCR products containing only Cj0859c were obtained from 84 strains. Both PCR product sizes were amplified using the same pg06.14 and pg06.15 primers. The deduced amino acid sequences of both genes were analyzed separately.

### Analysis of the deduced amino acid sequences of FspA

**Figure 1:**
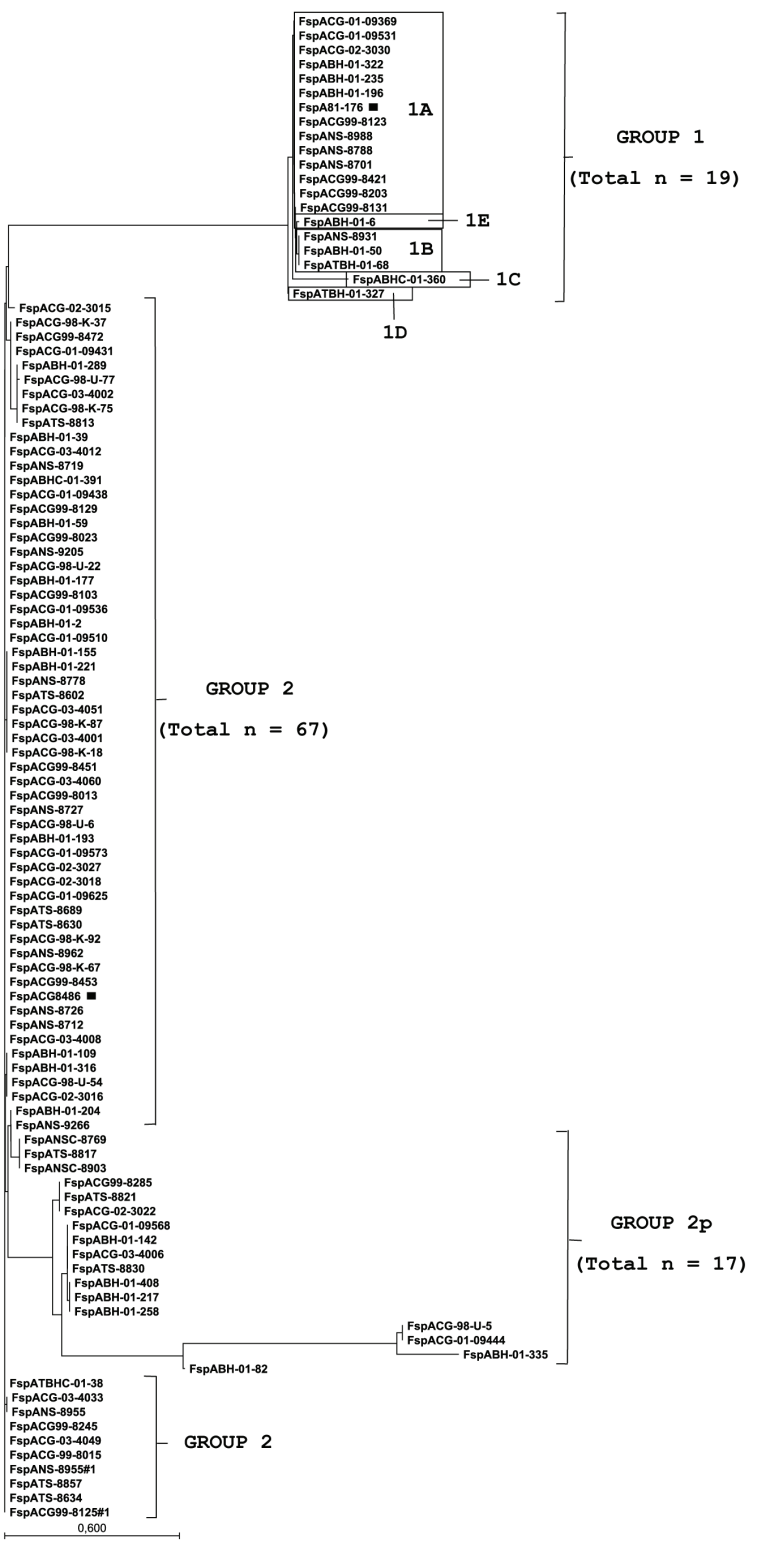
**Neighbor-joining tree based on the deduced amino acid sequences *fspA *of the 103 *C.jejuni *strains**. A bootstrap test was performed with 1,000 resamplings and scale bar represent 0.600 substitution per amino acid position.

**Figure 2:**
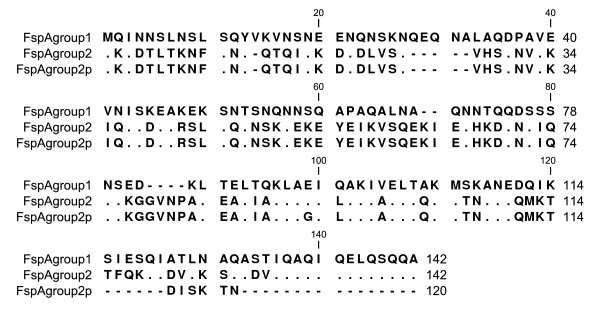
**Alignment of FspA group 1, 2 and 2p consensus sequences**.., identical amino acids; -, gaps.

The phylogenetic tree of predicted FspA sequences is shown in Figure 1. Twenty one amino acid patterns were found that clustered to 3 groups of FspA. FspA group 1 [GenBank:HQ909220 to: HQ909238] was similar to FspA1 as previously reported [12] and comprised 18.4% (19/103) of the total strains and consisted of 5 subgroups. All 5 subgroups were derived from only the 1600-bp PCR product that also contains an adjacent integral membrane protein gene as well. All subgroups in group 2 and 2p were derived from only the 800-bp PCR product. FspA group 2 [GenBank:HQ909136 to: HQ909202] was similar to FspA2 in the previous report [12]. FspA group 2 consisted of 9 subgroups and was the largest group in this study (65%, 67/103). In contrast, *fspA1 *(69.5%) was significantly more common than *fspA2 *(30%) among Finnish human strains [15]. FspA group 2p [GenBank:HQ909203 to: HQ909219] comprised 16.5% (17/103) of the total strains and consisted of 7 subgroups. Each subgroup of group 2p consists of 1-4 strains. FspA group 2p sequences were similar to FspA group 2 sequences (Figure 2), however FspA group 2p sequences were found to contain a stop codon at a position that before the terminal 142 position. Consequently, the predicted FspA group 2p amino acid sequences were shorter than the FspA group 2 sequences. As a result, FspA group 2p was treated as a novel group. Further studies are needed to determine if these sequences are expressed as malfunctioning end product of FspA. In conclusion, our study reveals greater heterogeneity of FspA (group 1, 2 and 2p) among Thai *C. jejuni *isolates than a previous report that found only FspA2 in Thailand [12].

### Alignment of the deduced amino acid sequences of FspA

Each subgroup in FspA group 1 was aligned and compared with reference sequence, *C. jejuni *81-176 (Table 1). Percent identities of the alignment with reference sequence ranged from 82% (117/142) to 100% (142/142). The most common amino acid sequence pattern (100% identity), classified as subgroup 1A was observed from 13 *C. jejuni *isolates of this group of 19 isolates. There were amino acid changes in subgroup 1B, 1D and 1E sequences, whereas internal deletions were found in subgroup 1C. For FspA group 2, each subgroup was aligned and compared with reference sequence, *C. jejuni *CG8486. Percent identities of the alignment with the reference sequence ranged from 95% (135/142) to 100% (142/142). The most common amino acid sequence pattern (100% identity), classified as subgroup 2A was observed from 42 *C. jejuni *isolates of this group (67 isolates). There were amino acid changes in sequences of subgroup 2B to 2I. Each subgroup of FspA group 2p was aligned and compared with reference sequence, *C. jejuni *CG8486. The percent identities of the alignment with reference sequence ranged from 25% (35/142) to 95% (135/142). Amino acid changes and deletion points were found within all subgroups in FspA group 2p.

**Table 1:** Distribution and alignment of the deduced amino acid sequence *fspA *of 103 *C. jejuni *strains

Group	Subgroup	Representative strain	Frequency	Alignment with reference C. jejuni*		
				
				Amino acid change†	Deletion point‡	Identity (%)
1	1A	CG-01-09369	13	-		142/142(100%)

	1B	NS-8931	3	N79D		141/142(99%)

	1C	BHC-01-360	1	-	37-61	117/142(82%)

	1D	TBH-01-327	1	S18L, D75N		140/142(98%)

	1E	BH-01-6	1	T53A		141/142(99%)

2	2A	BH-01-39	42	-		142/142(100%)

	2B	CG-03-4033	2	V33I		141/142(99%)

	2C	BH-01-316	4	E136K		141/142(99%)

	2D	BH-01-155	8	M112A		141/142(99%)

	2E	CG-98-K-37	3	N75S, V80G, K124I		139/142(97%)

	2F	BH-01-204	2	E93G, V129I, A142T		139/142(97%)

	2G	CG-02-3015	1	K8T, H28R, T47A, N75S, G78D		137/142(96%)

	2H	BH-01-289	4	D20N, V24A, T47A, N75S, V80G, K124I		136/142(95%)

	2I	CG-98-U-77	1	D20N, V24A, T47A, N75S, V80G, I88M, K124I		135/142(95%)

2p	2pA	NSC-8769	3	E93G, V129I, A142T	94-97	135/142(95%)

	2pB	CG99-8285	3	Q119T, I120N	121-142	118/142(83%)

	2pC	CG-01-09568	4	E93G, T115D, F116I, Q117S, Q119T, I120N	121-142	114/142(80%)

	2pD	BH-01-408	3	V80I, E93G, T115D, F116I, Q117S, Q119T, I120N	121-142	113/142(79%)

	2pE	BH-01-82	1	Q70K, L95P, A96F, E100K, L101V	80-93, 103-142	83/142(58%)

	2pF	CG-98-U-5	2	K58Q, V59M, S60K, K63V	38-57, 64-142	39/142(27%)

	2pG	BH-01-335	1	D20N, V24A, K58F, V59R	38-57, 60-142	35/142(25%)

*Group 1 was compared with *C. jejuni *81-176 whereas Group 2 and 2p were compared with *C. jejuni *CG8486.

†Standard single letter amino acid code and protein residue numbering are used.

‡Protein residue numbering is used.

For FspA group 1, percent identity within the group without reference sequence was 97.18%, except for strain BHC-01-360. The BHC-01-360 strain was aligned with *C. jejuni *81-176 and showed 25 deleted amino acids. The percent identity within FspA group 2 without reference sequence was 88.73%. The results imply that FspA group 1 is more conserved than FspA group 2. This is in contrast to the results in preceding report that FspA1alleles were less conserved than FspA2 [12].

The FspA group 1 consensus sequence encoded a predicted soluble cytoplasmic protein of 15.5 kDa, pI 4.84, and the FspA group 2 consensus sequence encoded a predicted soluble cytoplasmic protein of 16.0 kDa, pI 5.96. Additionally, the two variants were only 40.14% identical at the protein level (Figure 2). Therefore, FspA group 1 is obviously distinguishable from FspA group 2, as reported previously.

### Analysis of the deduced Cj0860 protein sequences

**Figure 3:**
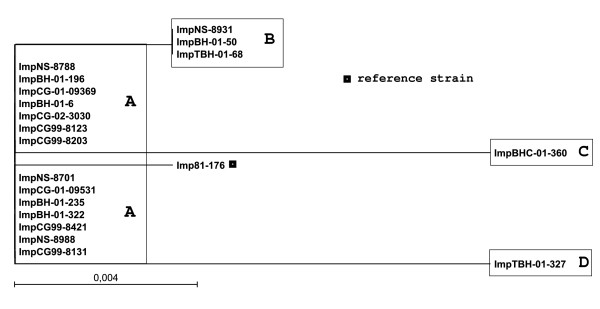
**Neighbor-joining tree based on the deduced integral membrane protein sequences of the 19 *C.jejuni *strains**. A bootstrap test was performed with 1,000 resamplings and scale bar represent 0.004 substitution per amino acid position.

Predicted protein sequences encoded by the Cj0860 alleles were derived from the 1600-bp PCR products that contain *fspA *group 1 gene as well. The integral membrane protein [GenBank:HQ909239 to: HQ909257] was found in only 18.4% (19/103) and clustered to 4 groups. Phylogenetic analysis of deduced integral membrane protein sequences was carried out as shown as Figure 3.

### Alignment of the deduced Cj0860 protein sequences

Each group was aligned and compared with reference sequence, *C. jejuni *81-176. Percent identities of the alignment with reference sequence ranged from 98% (286/290) to 99% (289/290). The most common amino acid sequence pattern (99% identity) was observed from 14 *C. jejuni *isolates of total 19 isolates. Amino acid changes were present in all groups. The analysis of alignment is summarized in Table 2. Percent identity within the group without reference sequence was 97.59%.

**Table 2:** Distribution and alignment of the deduced integral membrane protein sequence of 19 *C. jejuni *strains

Group	Representative strain	Frequency	Alignment with *C. jejuni *81-176	
			
			Amino acid change*	Identity (%)
A	CG-01-09369	14	I33V	289/290 (99%)

B	NS-8931	3	I33V, V289A	288/290 (99%)

C	BHC-01-360	1	I33V, V145I, G216S, V274A	286/290 (98%)

D	TBH-01-327	1	I33V, A52T, T59A, M69I	286/290 (98%)

*Standard single letter amino acid code and protein residue numbering are used.

### Association between subgroups of *fspA *group 1 and Cj0860 alleles

As reported previously, only *fspA *group 1 alleles were associated with Cj0860 alleles. Furthermore, each subgroup of FspA group 1 was associated with each group of integral membrane protein. Subgroup 1A of FspA group 1 included 1E that has only one amino acid different from 1A, were associated with group A of integral membrane protein. Subgroup 1B, 1C and 1D of FspA group1 were associated with group B, C and D of integral membrane protein, respectively. The associations are shown in Figure 1 and 3.

It has been known that only FspA group 1 encoding gene was adjacent to integral membrane protein gene. Moreover, each subgroup of FspA group 1 is linked with a specific allele of the integral membrane protein. This may imply that integral membrane proteins relate to virulence of FspA or that the two proteins co-evolved from a common precursor. However, this remains to be determined and more information about the functional association of the two proteins remains lacking.

## Conclusions

This study reveals greater heterogeneity of FspA (group 1, 2 and 2p) among Thai *C. jejuni *isolates than previously reported [12]. This may be because the selected *C. jejuni *isolates for this study were representative isolates from of PFGE clusters of isolates from multiple diarrhea studies in Thailand [16]. Moreover, these isolates were obtained from various regions of Thailand over time suggesting spatial and temporal distribution among *C. jejuni *isolates in Thailand, whereas all previously examined Thai isolates were from the 1999 Cobra Gold exercises [12]. Interestingly, the association of only the FspA group 1 gene with the Cj0860 alleles that was reported previously continued to be found in these additional strains. Moreover, subgroups of FspA group 1 were associated with specific groups of Cj0860. However, the significance of the observed differences and the roles of these genes in virulence need further study.

## Methods

### Bacterial strains

Of three hundred and thirty four *C. jejuni *isolates, 274 isolates were from US soldiers with diarrhea who participated in the Cobra Gold exercises during 1998-2003 and 60 isolates were from foreigners and Thai adults with diarrhea seen at Bumrungrad Hospital in 2001-2002. The 334 isolates had been previously clustered by PFGE analysis. A dendrogram of PFGE patterns was generated and grouped into 103 clusters. A total of 103 *C. jejuni *isolates representative of each cluster from the PFGE analysis were selected for this study [16]. Among 103 *C. jejuni *isolates, 76 isolates were isolated from the US soldiers in the Cobra Gold exercises and 27 isolates were isolated from the foreigners and Thai adults at Bumrungrad Hospital in Bangkok. All *C. jejuni *isolates were kept at -70 °C in glycerol medium and later were subsequently grown on blood agar plate (BD Diagnostic Systems, Sparks, MD, USA.) and incubated at 37 °C for 48 h under microaerobic condition. All isolates were from stool samples collected with informed consent under human use protocols approved by the appropriate ethical review committees.

### PCR of alleles of Cj0859c (*fspA) *and Cj0860

DNA templates were extracted from fresh, subcultured *C. jejuni *isolates after 48 h incubation using DNeasy tissue kits (Qiagen GmbH, Hilden, Germany). The samples of DNA were estimated for purity and quantity by gel electrophoresis compared to the known molecular weight marker and these DNA were kept at -20 °C for further use as DNA template for PCR. The *fspA *gene (Cj0859c) and the adjacent integral membrane protein gene (Cj0860), if present, were amplified using 0.4 μM of primer pg06.14 (5' -CCTATTTATGGATTGCAATTTCACCCCG -3') that bound to *pabA *gene (Cj0861) and 0.4 μM of primer pg06.15 (5' -CTTGAAACGATCAAGGGTAGGGCAGC -3') that bound to *murA *(Cj0858c) [12] in 50-μl reactions containing 1-10 ng DNA template, 1 × PCR buffer II, 3.0 mM MgCl_2_, 0.4 mM each dNTP and 2.5 U of AmpliTaq Gold DNA Polymerase (Applied Biosystems). DNA amplification was performed using an initial denaturation step at 94 °C for 5 min; followed by 35 cycles of amplification (denaturation at 94 °C for 1 min, annealing at 55 °C for 1 min, and extension at 72 °C for 1 min) and ending with a final extension at 72 °C for 10 min. Amplification products were purified for sequencing by Wizard SV gel and PCR clean-up system (Promega, WI, USA).

### Sequencing of PCR products and analysis of sequences

All amplified products were submitted for sequencing commercially (Macrogen, Seoul, South Korea). An approximately 800-bp PCR products were sequenced using the two PCR primers (pg06.14 and pg06.15) as previously described, whereas 1600-bp PCR products were sequenced using the two PCR primers (pg06.14 and pg06.15) and primer pg0553re (5'- GCTATTTAAGGAATTGTTAATTTGCAT-3'). The DNA sequences were edited and assembled by Sequencher software version 4.7 (Gene Codes Corporation, MI, USA). The deduced amino acid sequences were analyzed for their heterogeneity such as alignment, clustering and sequence information using CLC Free Workbench 4 software [17]. *C. jejuni *81-176 [GenBank:CP000538] and CG8486 [GenBank:EF058232] were used as reference sequences.

## Competing interests

The authors declare that they have no competing interests.

## Authors' contributions

PP performed the PCR and carried out the study project (including the data analysis and preparation of the draft manuscript), OS designed the study and revised manuscript critically for important intellectual content, LB provided ideas and comments on the draft manuscript, FP provided primer sequences and constructive discussion of the draft manuscript, PG provided comments and expertise on the molecular biology of *C. jejuni *and CJM conceived the idea for the study, worked on the final manuscript and gave final approval of the version to be published. All authors read and approved the final manuscript.
